# Emerging Clues of Regulatory Roles of Circular RNAs through Modulating Oxidative Stress: Focus on Neurological and Vascular Diseases

**DOI:** 10.1155/2021/6659908

**Published:** 2021-03-01

**Authors:** Lingfei Li, Zhumei Ni, Xiaoli Si, Lin Jiang, Hongfei Sang, Wenqing Xia, Zhenzhen Chen, Jinyu Huang, Jingfen Jin, Anwen Shao, Congguo Yin

**Affiliations:** ^1^Department of Neurology, Affiliated Hangzhou First People's Hospital, Zhejiang University School of Medicine, Hangzhou, China; ^2^The Fourth Clinical Medical College, Zhejiang Chinese Medicine University, Hangzhou, China; ^3^Department of Neurology, The Second Affiliated Hospital, School of Medicine, Zhejiang University, Hangzhou, China; ^4^Department of Hematology, Affiliated Hangzhou First People's Hospital, Zhejiang University School of Medicine, Hangzhou, China; ^5^Department of Cardiology, Affiliated Hangzhou First People's Hospital, Zhejiang University School of Medicine, Hangzhou, China; ^6^Department of Nursing, The Second Affiliated Hospital, College of Medicine, Zhejiang University, Hangzhou, Zhejiang, China; ^7^Department of Neurosurgery, The Second Affiliated Hospital, School of Medicine, Zhejiang University, Hangzhou, China

## Abstract

Circular RNAs (circRNAs) are novel noncoding RNAs that play regulatory roles in gene expression. Dysregulation of circRNAs is associated with the development and progression of several diseases, such as diabetes mellitus, nervous system diseases, cardiovascular diseases, and cancer. CircRNAs functionally participate in cell physiological activities through various molecular mechanisms. However, these molecular mechanisms are unclear. Oxidative stress is an essential factor in the pathogenesis of various diseases, including neurological diseases. Emerging roles of circRNAs have been identified in different systems in response to oxidative stress. In this review, we summarize the current understanding of circRNA biogenesis, properties, expression profiles, and the clues indicating the regulatory roles of circRNAs through oxidative stress in various systems, especially the nervous system.

## 1. Introduction

Circular RNAs (circRNAs) are a novel type of noncoding RNAs [1]. Instead of having a 5′ cap and 3′ tail structure like linear RNAs, circRNAs have a covalent loop structure, which is stable and evolutionally conserved [2, 3]. They are diverse and widespread throughout different eukaryotic cells [3]. One of the prominent roles of circRNAs is to regulate gene expression, and thus, they modulate progression in various diseases such as diabetes, nervous system diseases, cardiovascular diseases, and cancer [4, 5]. Molecular mechanisms of gene expression regulation by circRNAs include microRNA (miRNA) sponge effect, posttranscriptional regulation, and translation regulation [6, 7]. Notably, the progression of neurovascular diseases, one of the most prevalent and devastating diseases affecting adults worldwide [8], is linked to circRNAs widely associated with various pathological responses, including atherosclerosis, neuroinflammation, apoptosis, and neurogenesis [9–11]. Moreover, these circRNAs can be diagnostic, monitoring, therapeutic, and/or prognostic tools in the management of several diseases [12]. Oxidative stress refers to the pathological process of excessive production of reactive oxygen species (ROS) and/or weakened antioxidant capacity of the organism, resulting in an imbalance between ROS production and clearance and, consequently, accumulation of ROS in the body and oxidative damage of cells. This pathological process is closely related to neurovascular diseases. Various risk factors of neurovascular diseases, such as smoking [13], fluctuation of blood glucose levels [14], hypertension [15], and hyperhomocysteinemia [16], can all increase ROS production. Accumulating evidence indicates that circRNAs are involved in the process of oxidative stress [17] and may be an important mechanism linking oxidative stress-inducing pathological factors to associated pathological conditions of neurological diseases. Exploration of circRNAs and their correlated effects in neurovascular diseases may facilitate accurate diagnosis and serve as new therapeutic targets for the disease. Therefore, we reviewed the emerging clues of the regulatory role of circRNAs through oxidative stress in neurovascular systems.

## 2. CircRNAs: Discovery, Features, Biogenesis, and Classification

**Figure 1:**
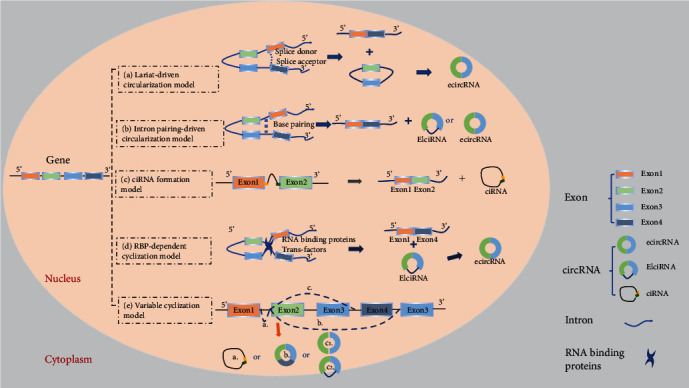
Schematic diagram illustrating five models of circRNA biogenesis. (a) Lariat-driven circularization model: the 3′ splice donor of exon 1 and the 5′ splice acceptor of exon 4 link up end-to-end by exon skipping and form an exon-containing lariat structure. Finally, the ecircRNA forms after introns are removed. (b) Intron pairing-driven circularization model: direct base pairing of introns forms a circulation structure, thereby forming ecircRNA or EIciRNA after intron removal. (c) ciRNA formation model: the elements near the splice site escape debranching stably so that the intron lariat is formed from the splicing reaction. (d) RNA binding protein- (RBP-) dependent cyclization model: RBPs bridge two flanking introns close together and then remove introns to form circRNAs. (e) Variable cyclization model: splicing selection and exon circularization may be influenced by inverted repeated Alu pairs (IRAlus) and the competition between them.

The presence of circRNAs in the cytoplasm of eukaryotic cells was observed under an electron microscope in the 1970s [18, 19], and later, circular transcripts of the testis-determining gene *Sry* were found in adult mice [20]. However, at that time, circRNAs were only considered products of missplicing, so their functions were underestimated [21]. With the help of high-throughput sequencing technology, a large number of circRNAs have been discovered and characterized [3]. The expression profile of circRNAs presents with three characteristics: (i) abundance: circRNAs are widely found in different cell types and organisms, such as *Caenorhabditis elegans*, *Drosophila*, mice and monkeys [22–24], plants [25, 26], and even protists [27–29]; (ii) stability: without 5′-3′ polarity and the covalent closed-loop structure of polyadenylate, circRNAs have high resistance to RNA exonuclease or ribonuclease R [30, 31]. Moreover, circRNA isomer transcripts have a half-life of more than 48 h; therefore, they are more stable than the related linear transcripts with a half-life of less than 20 h [3]; and (iii) conservation: circRNAs are evolutionarily conserved among humans, mice [11, 32, 33], and other mammals [34, 35] and even in insects [29, 32, 34]. Nowadays, it is realized that circRNAs are purposefully synthesized and generated by backsplicing, which is covalently linking free 3′ and 5′ ends [2, 36–40]. Based on the different proportion of exons and introns in the structure, circRNAs can be classified into three categories: exonic circRNAs (ecircRNAs) [3, 6, 32, 41], circular intronic RNAs (ciRNAs) [41], and exon-intron circRNAs (EIciRNAs) [42]. Five hypothesized models of circRNA biogenesis are illustrated in Figure 1: (i) lariat-driven circularization model: during heterogeneous nuclear RNA transcription, the RNA partially folds close to the nonadjacent exon, which leads to an exon skipping event, which results in covalent splices from the 3′ end of a splice donor to the 5′ end of a splice acceptor, forming an exon-containing lariat structure. Afterwards, the introns are removed, thus forming an ecircRNA (Figure 1(a)) [3]; (ii) intron pairing-driven circularization model (also known as “direct backsplicing”) [39]: alternative splicing to a circular molecule is presumably based on the pairing of complementary motifs in the transcripts, which may bring two exons closer to each other, resulting in an ecircRNA or EIciRNA after removing or retaining certain introns (Figure 1(b)) [3, 5]; (iii) ciRNA formation model: this is a novel model that occurs due to failure in debranching, which depends on a 7-nucleotide (nt) guanine uracil-rich element near the 5′ splice site and an 11-nt cytosine-rich element near the branch point (Figure 1(c)) [37, 41]; (iv) RNA binding protein- (RBP-) dependent cyclization model: by the interaction between RBPs, flanking introns in RNA molecules can be bridged causing the formation of circRNAs. Muscleblind (MBL) proteins and quaking proteins are two typical RBPs to mediate the circularization (Figure 1(d)); and (v) variable cyclization model: a single gene locus could produce multiple circularized exons. Competition of RNA pairing within individual introns or across flanking introns can alter exon circularization, which is evolutionarily dynamic (Figure 1(e)) [43].

## 3. Molecular Mechanisms of circRNAs in Gene Expression Regulation

### 3.1. CircRNAs Act as a Sponge for miRNAs or Competitive Endogenous RNAs (ceRNAs)

**Figure 2:**
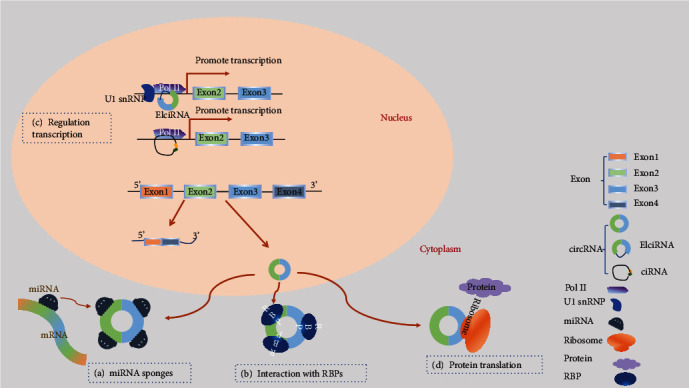
Schematic diagram illustrating functions of circRNAs. (a) Acting as miRNA sponges: circRNAs contain a common miRNA response element (MRE) that can bind to miRNA and prevent them from interacting with mRNA. (b) Interaction with RBPs: circRNAs can bind to RBPs to regulate mRNA expression by altering the splicing pattern or mRNA stability. (c) Regulation transcription: circRNAs play a regulatory role in the transcription of their parent coding genes. (d) Protein translation: circRNAs have coding potential and can be translated into proteins with ribosomes.

Several miRNA response elements are involved in mediating circRNAs competitively binding to miRNAs. CircRNAs are thus defined as miRNA sponges as they can “absorb” (i.e., binding to) miRNAs (Figure 2(a)) [7]. They regulate gene expression through binding to miRNAs and inhibit miRNA binding to messenger RNA (mRNA) in the cytoplasm [7, 44]. A pioneer study by Hansen et al. demonstrated that the circRNA ciRS-7 has a highly enriched miRNA binding site for miR-7 [7], which is a microRNA involved in numerous pathways and diseases [45, 46]. They also discovered that circRNAs from the mouse *Sry* gene have 16 putative target sites for miR-138 and work as sponges for this miRNA [7]. Recently, increasing evidence demonstrated that circRNAs work as sponges to regulate gene expression in neurovascular diseases. CircGRM4 acts as a sponge and is significantly increased in the eyes of cystathionine *β*-synthase-deficient mice. This results in the excessive turnover of the metabotropic glutamate receptor 4 in response to extremely high levels of circulating glutamate which mediates neurovascular toxicity [46]. However, a recent study showed that most circRNAs may not function as miRNA sponges [47]. Therefore, it is possible that the interactions between circRNAs and miRNAs are not simply inhibition-related but related to storage, sorting, and localization of miRNAs [48].

### 3.2. CircRNAs Interact with RBPs and mRNAs

In addition to the interaction with miRNAs, circRNAs also have a mutual effect on RBPs, including binding, storing, or sequestering RBPs to subcellular locations and acting as competing elements (Figure 2(b)). In 2013, circMBL was demonstrated to bind to MBL resulting in competition with canonical linear splicing and regulation of genes [49]. CircRNA poly(A) binding protein nuclear 1 (circPABPN1) can bind to human antigen R (HuR) and prevent HuR binding to PABPN1 mRNA, thus affecting translation. This is the first example of competition between a circRNA and its cognate mRNA for an RBP that affects translation [50]. In the same year, Yang et al. found that circAmotl1 can enhance nuclear translocation of the signal transducer and activator of transcription 3 to promote its interaction with the DNA (cytosine-5)-methyltransferase 3A (Dnmt3a) promoter and facilitate the transcription of Dnmt3a [51]. Oxidative stress may also be regulated by circRNAs through interactions with RBPs. circRNA forkhead box O3 (circFoxo3) could bind to cell cycle-associated proteins, cyclin-dependent kinase 2 and p21, which block the transition from G1 to S phase, therefore repressing cell proliferation and cell cycle progression [52]. Furthermore, circFoxo3 can suppress the antisenescence and antistress roles of inhibitors of DNA binding, E2F transcription factor 1 (involved with regulation of retinoblastoma and glioblastoma multiforme), focal adhesion kinase, and hypoxia-inducible factor-1*α*, by binding to them [53]. In addition, circRNAs can regulate the expression of linear protein-encoding RNA products by “mRNA trap” mechanisms.

### 3.3. Regulation of Transcription or Alternative Splicing

Recent advances have reported a *cis*-regulatory role of circRNAs on their parent coding genes (Figure 2(c)). CiRNA ankyrin repeat domain 52 (ci-ankrd52) largely accumulates in the elongating polymerase II (Pol II) complex and interacts with it to activate transcription of its parent gene. The expression of *ANKRD52* mRNA was reduced when ci-ankrd52 was knocked down [41]. Subsequently, researchers also found EIciRNAs, such as circRNA eukaryotic translation initiation factor 3 subunit J (circEIF3J) and circRNA poly(A) binding protein interacting protein 2 (circPAIP2), predominantly localized in the nucleus. EIciRNAs can interact with the U1 small nuclear ribonucleoproteins (snRNPs) by specific RNA-RNA interactions between U snRNA and EIciRNAs, leading to increased interactions between the EIciRNA-U1 snRNP complexes with Pol II and enhancing transcription of their parental genes [42]. Additionally, another study uncovered that circRNAs can reduce protein expression by sequestering the translation start site and producing noncoding linear transcripts [39].

### 3.4. Protein Translation

Apart from the abovementioned functions, circRNAs are also involved in protein translation (Figure 2(d)). In 2017, it was confirmed that the endogenous circRNA zinc finger protein 609 (circZNF609) can encode proteins. CircZNF609 functions in murine and human myoblasts and can be translated into a protein in a splicing-dependent and cap-independent way [54]. Pamudurti et al. demonstrated that circRNAs generated from the *MBL* locus have protein-encoding capacity and allow cap-independent translation [34]. Furthermore, circRNA F-box and WD repeat domain containing 7 (circFBXW7) can translate short and stable FBXW7-185 amino acid, which is a novel protein playing a definitive tumor-suppressive role and has potential prognostic significance in brain cancer [55]. Evidence has also been found in neurological diseases that circSHPRH (SNF2 histone linker PHD RING helicase) encodes SHPRH, a novel protein of 146 amino acids, which extends patient survival time in glioblastoma patients [56].

## 4. The Roles of circRNAs in Regulating Neurovascular Diseases

The first study investigating the role of circRNAs in ischemic stroke was done by Mehta et al. in 2017. They found significant changes in the expression of circRNAs in the ischemic penumbral cortex in mice with transient middle cerebral artery occlusion (tMCAO). circRNA expression profiles in the penumbral cortex at 6, 12, and 24 h after reperfusion were investigated. There were 283 circRNAs altered in tMCAO mice compared with those in the sham group, and 16 of the 283 circRNAs changed at all three time points. These results indicated the possible functional implication of circRNAs in poststroke pathophysiology [57]. Dou et al. explored the expression of circRNAs in rat brains at 6, 12, and 24 h after the onset of intracerebral hemorrhage (ICH) using microarray analysis. There were 93 and 20 circRNAs that were upregulated and downregulated, respectively, at all times compared to those in the sham group. The authors designed a network of circRNA-miRNA-mRNA bioinformatic methods and found that circRNAs can reduce brain damage, attenuate neurological dysfunction, and improve prognosis after ICH, exposing a potential possibility of circRNAs as a therapeutic target for ICH [58]. In addition, Han et al. also observed the upregulation of circRNA HECT domain E3 ubiquitin-protein ligase 1 (circHECTD1) in the tMCAO mouse models by microarray analysis, which was validated in patients with acute ischemic stroke (AIS) [59].

CircHECTD1 was shown to serve as a microRNA-142 sponge to inhibit the expression of TCDD-inducible poly(ADP-ribose) polymerase expression, thus inhibiting the activation of astrocytes. Taken together, circHECTD1 also can be a novel biomarker of stroke [59]. CircDLGAP4 was increased in both a mouse stroke model and the plasmas of 13 female and 13 male patients [60]. It acts as an miRNA-143 sponge, thereby inhibiting miR-143 activity and reducing HECTD1 expression. Importantly, the overexpression of circDLGAP4 protected nerves and alleviated blood-brain barrier damage in one study, making it a potential novel therapeutic target for the treatment of acute cerebral ischemia [60]. In China, researchers found 3 circRNAs (circFUNDC1, circPDS5B, and circCDC14A) which were significantly upregulated and positively correlated with infarct volume in the plasma of patients with AIS compared with healthy controls. The combination of these circRNAs has high specificity and sensitivity in the diagnosis of AIS [61]. As an important clinical pathological process, cerebral ischemia-reperfusion injury (IRI) is of great research value. Lin et al. established a model of oxygen-glucose deprivation/reoxygenation (OGD/R) in HT22 cells and compared the circRNA expression profile to normal controls by using circRNA microarrays. The results revealed the upregulation of 3 circRNAs and the downregulation of 15 circRNAs. By quantitative real-time PCR and bioinformatic analysis, a particular circRNA, mmu-circRNA-015947, was significantly upregulated and interacted with microRNAs to enhance target gene expression [62]. Another study analyzing the expression of circRNAs 24 h after reperfusion observed higher expression levels of circ_008018 in the cortical tissue of tMCAO mice than in the sham group. Notably, inhibition of circ_008018 can induce overexpression of miR-99a, thereby reversing the decrease of AKT phosphorylation and glycogen synthase kinase 3b caused by IRI to protect brain tissue [63]. Atherosclerotic plaque rupture is an important pathogeny of AIS. In one study, circR-284, which has miR-221 and miR-222 binding sites to regulate the activity of these miRNAs, was expressed in human vascular smooth muscle cells (VSMCs). These miRNAs inhibit oxidative stress induced by oxidized low-density lipoprotein and are novel regulators for VSMC proliferation and neointimal hyperplasia [64–66]. The expression of miR-221 and miR-222 decreases acutely, accompanied by an increase in p27Kip1 after plaque rupture, suggesting they may have plaque-stabilizing capabilities [67]. Bazan et al. compared the expression of miR-221, miR-222, and circR-284 in serum between patients who suffered from an acutely symptomatic ischemic cerebrovascular event within the previous five days and asymptomatic patients. They found that the ratio of serum circR-284 to miR-221, which has the potential to detect plaque rupture and stroke, was significantly raised in the symptomatic group, and this result was verified in a study with 112 patients [64].

## 5. Regulatory Role of CircRNAs in Oxidative Stress: Implications in Neurovascular Diseases

ROS produced during oxidative stress causes oxidative damage to cells. ROS is increased in and associated with various risk factors of neurovascular diseases [13–16]. The roles of circRNAs in modulating oxidative stress have been discovered in both the basic and clinical studies (Table 1), and circRNAs are widely involved in different molecular mechanisms contributing to the pathophysiological processes of neurovascular disease [12, 68]. Therefore, circRNAs may play an important role in the pathophysiology of neurovascular diseases.

### 5.1. CircRNAs Regulate Neurovascular Diseases and Other Vascular-Related Diseases through Modulating Oxidative Stress Processes

**Figure 3:**
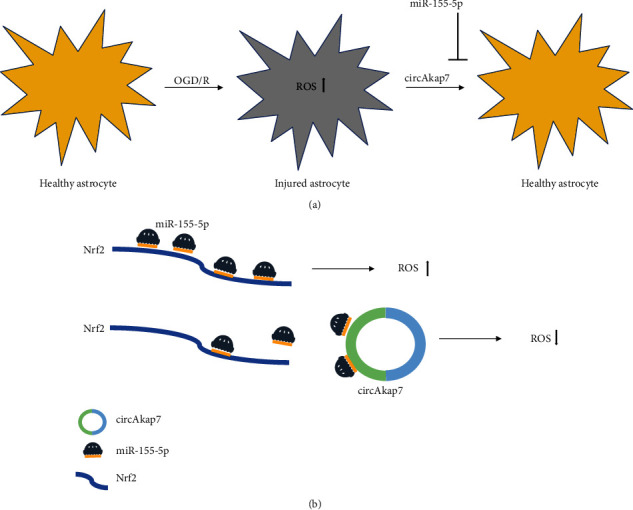
The regulatory role of circAkap7 via oxidative stress in a cell model of ischemic stroke. (a) The oxygen-glucose deprivation/reoxygenation (OGD/R) increases the reactive oxidative stress levels in primary astrocytes, whereas coculture with circAkap7 delivered by exosomes suppresses this increase. Moreover, circAkap7 is negatively regulated by miR-155-5p. (b) circAkap7 reduced OGD/R-induced cellular injury by absorbing miR-155-5p, which promotes Nrf2-mediated oxidative stress.

CircAkap7 was downregulated in a mouse model of tMCAO, which is a classic model for ischemic stroke. A high-throughput circRNA microarray study revealed that in tMCAO mice, mm9_circ_010383 (circAkap7) was suppressed [57]. A-kinase anchor protein 7 (AKAP7) is widely expressed throughout the brain, and the early heightened expression levels of *AKAP7* were significantly associated with the blood-brain barrier [69, 70]. A study by Xu and colleagues reported that exo-circAkap7 can reverse the significantly high expression of ROS and malondialdehyde (MDA) in the tMCAO group. After the treatment with exo-circAkap7, the levels of circAkap7, autophagy-related gene *ATG12* (Figure 3(a)), and oxidative stress-related gene *NRF2* were significantly increased via absorbing miR-155-5p [71] (Figure 3(b)). Up to now, there are a few papers showing the regulatory role of circRNAs in ischemic stroke through modulating oxidative stress. More studies should be designed to investigate this topic. Moreover, circRNAs have been found to be involved in vascular-related diseases. CircRNA zinc finger protein 609 (circZNF609) was abundantly expressed in endothelial cells [72] and upregulated under hypoxia stress in a diabetic retinopathy mouse model [73]. Silencing circZNF609 protected endothelial cells from hypoxia and oxidative stress *in vivo* by reducing capillary degeneration and pathological angiogenesis, as well as partially rescuing human umbilical vein endothelial cells (HUVECs) from oxidative stress- and hypoxic stress-induced cell apoptosis [73]. Moreover, the overexpression of miR-615-5p can also reduce diabetes mellitus-induced retinal vascular leakage and rescue aggravated capillary degeneration from oxidative stress and hypoxia-induced apoptosis. Taken together, these results suggested circZNF609 works through a signaling network which is composed of circZNF609 and miR-615-5p [73]. Atherosclerosis is a pathological process of ischemic stroke [74]. Reducing the expression of circANRIL (circular antisense noncoding RNA in the INK4 locus) can prevent the progression of atherosclerosis by reducing the levels of inflammatory factors and the serum levels of lipids, triglycerides, low-density lipoprotein, interleukin- (IL-) 1, IL-6, matrix metalloproteinase-9, and C-reactive protein and alleviating the apoptosis of vascular endothelial cells [75]. Similarly, another study reported that circANRIL may alter RNA function and confer atheroprotection. It can also promote nucleolar stress and p53 activation by combining with the pescadillo ribosomal biogenesis factor 1, thereby controlling the maturation of ribosomal RNA and inhibiting proliferation [9]. Shi et al. found that in patients with coronary heart disease, inhibiting circANRIL expression can reduce vascular endothelial injury, oxidative stress, and inflammation [76].

Liu et al. found that under oxidative stress, circHIPK3 (homeodomain interacting protein kinase 3) silencing increased the number of cell death, inhibited cell proliferation, and finally promoted the occurrence and development of apoptosis [77]. CircHIPK3 acts as a sponge for miR-193a-3p, the inhibitor of which significantly increases the viability and proliferation and rescues the effects of circHIPK3 silencing. It is obvious that circHIPK3 regulates the function of human lens epithelial cells *in vitro* through the circHIPK3/miR-193a-3p/crystallin alpha A network axis [77]. In the cardiac microvascular endothelial cells (CMVECs) under oxidative stress conditions (hydrogen peroxide- (H_2_O_2_-) induced), circHIPK3 can be transferred by exosomes released from CMVECs pretreated with hypoxia and can regulate oxidative damage in these cells via the miR-29a/IGF-1 axis. circHIPK3 overexpression significantly decreases apoptosis and protects CMVECs against H_2_O_2_-induced oxidative damage [78]. It is also reported that cardiac endothelial cells can internalize exosomes containing circHIPK3 and increase circHIPK3 levels causing inhibition of miR-29a activity, increasing VEGFA expression, and regulating angiogenesis [79]. In high glucose- (HG-) induced HUVECs, the expression of circBPTF (bromodomain PHD finger transcription factor) is upregulated. This regulates HG-induced HUVEC dysfunction, including apoptosis, inflammation, and oxidative stress through the miR-384/LIN28B axis. The circBPTF knockdown can increase LIN28B to enhance cell viability and prevent inflammatory damage and oxidative stress [80]. LIN28B is an RNA binding protein which represses oncogenes by inhibiting the formation of the let-7 family of miRNAs [81]. A study by Wang and colleagues revealed that HG-induced HUVEC exosomes (HUVEC-Exos) treated VSMCs. Lactate dehydrogenase levels were higher, and the activity of the antioxidative stress marker, superoxide dismutase (SOD), was lower, compared with that of VSMCs incubated with normal glucose HUVEC-Exos. These results suggested that HG-HUVEC-Exos with circRNA-0077930 can induce cellular oxidative stress and increase the expression of senescence-associated proteins (such as Kras, p21, and p53) via the circRNA-0077930-miR-622-Kras ceRNA network, causing the senescence of VSMCs [82].

### 5.2. CircRNAs Regulate Other Neurological Diseases via Modulating Oxidative Stress Processes

Neural tissues are sensitive to oxidative stress, so oxidative stress is one of the major mechanisms involved in neuronal damage and death after AIS [83–85]. Nuclear factor erythroid 2-related factor 2 (Nrf2) is a transcription factor regulating the transcription of antioxidant response elements [86]. It drives the expression of various detoxification and antioxidant factors, such as SOD1, glutamate-cysteine ligase regulatory subunit, GPx2, and glutathione reductase through Nrf2-related pathways [87], thus playing a neuroprotective role in oxidative stress associated with acute neuronal injury and chronic neurodegeneration. Yang et al. found that mmu-circRNA-32463 and mmu-circRNA-015216 may be potential regulators of Nrf2-mediated neuroprotection against oxidative stress [88]. CircSLC8A1 (solute carrier family 8 member A1), arising from the sodium-calcium exchanger gene *SLC8A1*, is a circularized exon 2 transcript [89]. As an Argonaute-2-bound RNA, it can also bind and may even modulate the activity of several miRNAs, including miR-128 and miR-133a [90]. In oxidative stress, oxidation itself may increase the circulation of circSLC8A1 in neurons and reduce the degradation of circRNAs, ultimately reducing the expression of the SLC8A1 protein. Inversely, in human neuroblastoma SH-SY5Y cells treated with statins (drugs with antioxidant potential), both circSLC8A1 and SLC8A1 protein are reduced [91]. Cheng et al. show that H_2_O_2_ downregulates the expression of circPRKCI (protein kinase C iota) and induces neuronal injury through the circPRKCI-miR-545/589-E2F2 axis. The overexpression of circPRKCI alleviates H_2_O_2_-induced cytotoxicity [92].

There are 15 circRNAs significantly changed in an oxygen-glucose deprivation/reoxygenation (OGD/R) model of cultured hippocampal HT22 cells, and the upregulated expression of mmu-circRNA-015947 indicated that it may be involved in the pathogenesis of cerebral IRI through the Kyoto Encyclopedia of Genes and Genomes pathway analysis [62]. Ding et al. found that the upregulation of miR-7a could regulate mitochondrial function and enhance the expression of antioxidases to alleviate the injury-induced oxidative stress in rats with spinal cord injuries [93]. CircRNA Cdr1as is derived from an antisense transcript of the *Cdr1* protein-coding gene at chromosome X (NC_000086.7) in mice [94] and functions as an miR-7 sponge in neuronal cells [7]; therefore, it can be speculated that circCDR1 may aggravate the oxidative stress in neuronal cells by acting as an miR-7 sponge [94].

Liu et al. discovered that the expression of circACR was reduced in HG-irritated rat Schwann RSC96 cells, which relieved the oxidative stress caused by HG via declining miR-145-3p and promoting PI3K/AKT/mTOR pathway activation [95].

### 5.3. CircRNAs Regulate Various Diseases via Modulating Oxidative Stress Processes

Apart from neurological diseases, regulatory roles of circRNAs through modulating oxidative stress are also found in other diseases, which are summarized in Table 1.

#### 5.3.1. Cardiomyocytes

Tian et al. found that atorvastatin can rescue cardiomyocytes from oxidative stress-induced apoptosis through decreasing microRNA-143 (miR-143) levels [96], and Bai et al. demonstrated that circDLGAP4 can inhibit miR-143 activity by acting as an endogenous miR-143 sponge [60]. In another study, Wang et al. found that the circDLGAP4/miR-143/BCL2 axis is a potential regulated axis in oxidative stress and myocardial IRI [97]. Upregulation of hsa_circ_0000064 (circ_0000064) weakened autophagy, reduced levels of lactate creatine kinase and dehydrogenase, inhibited oxidative stress, and significantly reduced myocardial infarction areas, signaling circ_0000064 had a protective effect against myocardial IRI [98]. Wang et al. found that acting as an miR-223 sponge, the heart-related circRNA could sequester and inhibit the activity of miR-223, which is confirmed as an important regulator of cardiomyocyte apoptosis under oxidative stress [99, 100]. In H9c2 (fetal cardiomyocyte-derived) cells, upon treatment with 100 *μ*M H_2_O_2_, it was observed that the circNCX1 level increased. Acting as an endogenous miR-133a-3p sponge, circNCX1 responds to the ROS increase which eventually leads to the expression of proapoptotic factor cell death-inducing protein, which ultimately leads to the death of cardiomyocytes *in vitro* and *in vivo* [101].

#### 5.3.2. Others

Li et al. reported that the knockdown of circRNA_0084043 may decrease MDA content and enhance activities of SOD and glutathione peroxidase, resulting in weakening the oxidative stress in HG-indicated human retinal pigment epithelium ARPE-19 cells [102]. It has been established that circAKT3 can regulate oxidative stress to promote renal IRI progression. In I/R+circAKT3 rats, the MDA and O_2_ levels were significantly increased and the SOD and catalase levels were significantly downregulated [103]. In the experiments performed by Yin et al., the knockdown of circSCAF11 upregulated cellular ROS levels and promoted 8-hydroxy-2-deoxyguanosine (8-OHdG) production in T98G and LN229 cells, which can be decreased by an miR-145-5p inhibitor [104].

Feng et al. revealed a crucial role for the circPRKCB/miR-339-5p/p66Shc signaling pathway in regulating oxidative stress in the I/R intestine. CircPRKCB can regulate p66Shc expression and hypoxia/reoxygenation- (H/R-) induced oxidative stress acting as an endogenous miR-339-5p sponge. The silencing of circPRKCB significantly inhibited the increased expression of intracellular ROS levels, mitochondrial O_2_ levels, and NOX2 and the decreased expression of H/R-induced manganese SOD and catalase [105].

Researchers found that in HaCaT cells treated with H_2_O_2_, which were considered a cell model of pressure ulcers, circZNF609 silencing may reduce oxidative stress by regulating miR-145 [106]. CircNFIX is abundant, conservative, and stable in H9c2 cells. In an anoxic environment, circNFIX expression was significantly downregulated in cardiomyocytes under oxidative stress [107]. Chen and colleagues found that the HG treatment can significantly increase ROS and MDA levels and decrease SOD activity and SOD2 expression. Moreover, they reported that circLRP6 regulates HG-induced proliferation, extracellular matrix accumulation, inflammation in mesangial cells, and oxidative stress via sponging miR-205, upregulating high mobility group box 1, and activating toll-like receptor 4/nuclear factor-kappa B signaling [108].

## 6. Perspectives

CircRNAs are potential pivotal biomarkers for diagnosis of diseases and prognosis because they are abundant, stable, and conserved. CircRNAs participate in regulating gene expression in various diseases through three molecular mechanisms: acting as a sponge of miRNAs or ceRNAs, interaction with RBPs and mRNAs, and regulation of transcription or alternative splicing. Increasingly, there is evidence to show that circRNAs are involved in the process of oxidative stress, which has been shown to be closely related to neurovascular diseases. Therefore, circRNAs may affect the occurrence and development of neurovascular diseases by regulating the oxidative stress process. In this review, the possible changes in circRNAs in cerebrovascular diseases and the results were described in detail, suggesting that circRNAs may be a biomarker of cerebrovascular diseases and a potential new therapeutic target. This article also summarizes that circRNAs regulate the occurrence and development of neurovascular diseases and other vascular-related diseases by regulating oxidative stress: first, under oxidative stress, circRNAs change and result in the development of diseases; second, the change in circRNAs may lead to oxidative stress and result in the occurrence and development of diseases. All evidence suggests that circRNAs may be important in the oxidative stress-induced pathophysiology of neurovascular diseases.

## Figures and Tables

**Table 1:** The evidence of regulatory roles of circRNAs in various diseases under oxidative stress.

*Evidence from basic research*						
*Cell line/animal*	*Disease model/treatment*	*Sample*	*circRNA*	*Alteration of expression*	*Various roles in the pathophysiological mechanism*	*Reference*
HT22 cells	OGD/R	Cell	mmu-circRNA-015947	Upregulated	May participate in apoptosis-related, metabolism-related, and immune-related pathways	[62]
CMVECs	Oxidative stress induced by H_2_O_2_	Cell	circHIPK3	Upregulated	Overexpression significantly decreased apoptosis and protected CMVECs against H_2_O_2_-induced oxidative damages	[78]
HUVECs	Oxidative stress induced by HG	Cell	circBPTF	Upregulated	Regulate HG-induced HUVEC dysfunction, including apoptosis, inflammation, and oxidative stress	[80]
SH-SY5Y cells	Oxidative stress induced by H_2_O_2_	Cell	circPRKCI	Downregulated	Alleviate the cytotoxicity induced by H_2_O_2_	[92]
ARPE-19 cells	Oxidative stress induced by HG	Cell	circRNA_0084043	Upregulated	May enhance malondialdehyde content and decrease activities of SOD and glutathione peroxidase, resulting in enhancing the oxidative stress in HG-indicated human retinal pigment epithelium ARPE-19 cells	[102]
Rat Schwann RSC96 cells	Oxidative stress induced by HG	Cell	circACR	Downregulated	Relieve the oxidative stress caused by HG	[95]
Vascular smooth muscle cells	Hyperglycemia stimulation	Exosomes	circRNA-0077930	(See the right column)	Its overexpression increases the lactate dehydrogenase activity, reduces the antioxidative stress marker (SOD) activity, and induces senescence	[82]
H9c2 cells	100 *μ*M H_2_O_2_ treatment	Cell	circNCX1	Upregulated	Lead to the death of cardiomyocytes *in vitro* and *in vivo*, acting as an endogenous miR-133a-3p sponge	[101]
H9c2 cells	Oxidative stress induced by H_2_O_2_	Cell	circNFIX	Downregulated	Facilitates oxidative stress-induced H9c2 cells apoptosis	[107]
Mesangial cells	High glucose- (HG-) treated	Cell	circLRP6	Upregulated	Regulates HG-induced proliferation, oxidative stress, ECM accumulation, and inflammation in mesangial cells via sponging miR-205, upregulating HMGB1, and activating TLR4/NF-*κ*B pathway	[108]
Rat	ICH	Cortical tissues around the hematoma	rno_circRNA_011054 rno_circRNA_005098 rno_circRNA_012556	Upregulated Downregulated	Promising targets for therapeutic intervention of ICH	[58]
Chicken	NH_3_ treatment	Thymuses	circRNA-SMC6	(See the right column)	Correlated with OXR1, which can protect cells from oxidative damage	[109]
Mouse	Nrf2 knockout	Substantia nigra	mmu-circRNA-32463 mmu-circRNA-015216	Upregulated Downregulated	A potential regulator of Nrf2-mediated neuroprotection against oxidative stress	[88]
Mouse	Nrf2 knockout	Corpus striatum	mmu-circRNA-017077	Downregulated	Participate in Nrf2-mediated Fas-induced apoptosis in a variety of neurological diseases associated with oxidative stress	[88]
Mouse	tMCAO	Cortical tissue	circ_008018	Upregulated	Result in brain tissue damage and neurological deficits	[63]
Mouse	Oxidative stress induced by H_2_O_2_	Cardiac endothelial cells	circHIPK3	Enriched in exosomes	Promote accelerated cell cycle progression and proliferation	[79]
Mouse	tMCAO	Cell lysates	circ_008018 circ_015350 circ_016128 circ_011137 circ_001729 circ_006696	Upregulated 6 hours reperfusion after transient MCAO Downregulated 6 hours reperfusion after transient MCAO	Might have functional consequences in controlling the secondary brain damage and neurological dysfunction	[57]
Mouse	tMCAO	Cell lysates	circDLGAP4	Downregulated	Decrease neurological deficits and infarct areas and blood-brain barrier (BBB) damage	[60]
Mouse	Diabetic retinopathy	Cell lysates	circZNF609	Upregulated	Capillary degeneration and pathological angiogenesis	[73]
Rat	Coronary atherosclerosis	Coronary artery tissue	circANRIL	In the empty vector and overexpressed cANRIL groups, atherosclerotic plaques and thrombi appeared	Reduced cANRIL expression could prevent coronary AS by reducing vascular EC apoptosis and inflammatory factor expression	[75, 76]
Mouse	Hypertrophy and heart failure induced by ISO treatment	Cardiomyocytes	mm9_circ_012559, circHRCR	Downregulated	Inhibits cardiac hypertrophy and heart failure by targeting miR-223 and ARC	[99]
Mouse	MI	Mouse myocardial tissues and mouse blood samples	circCDR1	Upregulated	May aggravate the oxidative stress in neuronal cells	[94]
Rat	Renal ischemia-reperfusion	Mouse kidney tissues	circAKT3	(See the right column)	Involve RI/R injury progression through regulating miR-144-5p/Wnt/*β*-catenin pathway and oxidative stress	[103]
Rats	I/R pretreatment with salidroside	Rat heart tissues	circ_0000064	Upregulated	Have a protective effect on myocardial ischemia-reperfusion injury	[98]

*Evidence from clinical research*						
*Disease*	*Sample*	*Treatment*	*circRNA*	*Alteration of expression*	*Various roles in the pathophysiological mechanism*	*Reference*
AIS	Plasma from patients	N/A	circDLGAP4	Downregulated	Decrease neurological deficits and infarct areas and blood-brain barrier (BBB) damage	[60]
AIS	Plasma from patients	N/A	circFUNDC1, circPDS5B, circCDC14A	Upregulated	Serve as biomarkers for AIS diagnosis and prediction of stroke outcomes	[61]
Acute carotid-related cerebrovascular ischemia	Plasma from patients	N/A	The ratio of circRNA-284/miR-221	Increased	Detect plaque rupture and stroke, as biomarkers in cardiovascular disease	[64]
Parkinson's disease	Postmortem brain tissues from patients	N/A	circSLC8A1	Upregulated	In oxidative stress, oxidation itself may increase the circulation of circSLC8A1 in neurons and reduce the expression of SLC8A1 protein	[91]
Glioma	In glioma tissues from patients and cells (T98G and LN229)	N/A	circSCAF11	Upregulated	Its knockdown repressed cell proliferation and invasion	[104]
Intestinal infarction	In the intestinal mucosal tissues of mice and patients undergoing surgery for acute mesenteric arterial embolism, strangulated intestinal obstruction, or incarcerated hernia	I/R	circPRKCB	Upregulated	circPRKCB silencing or miR-339-5p overexpression significantly downregulated p66Shc expression and attenuated oxidative stress levels and I/R injury	[105]
Atherosclerotic plaques	SMCs and CD68-positive macrophages from patients	N/A	circANRIL	Upregulated	May alter RNA function and confer atheroprotection	[9]
ARC	Lens epithelial cells from patients	N/A	circHIPK3	Downregulated	Under oxidative stress, circHIPK3 silencing increased the number of cell death and inhibited cell proliferation	[77]
Human dental pulp stromal cells (a mesenchymal stromal cell source for regenerative therapy)	Human dental pulp stromal cells	Oxidative stress induced by H_2_O_2_	hsa_circ_0000257	Upregulated	The hsa_circ_0000257/hsa-miR-9-5p/SIRT1/p53 regulatory axis is likely a novel molecular pathway regulating oxidative stress in hDPSCs	[110]

Abbreviations: AIS: acute ischemic stroke; ARC: age-related cataract; CMVECs: cardiac microvascular endothelial cells; H_2_O_2_: hydrogen peroxide; HUVECs: human umbilical vein endothelial cells; ICH: intracerebral hemorrhage; I/R: ischemia-reperfusion; MI: myocardial infarction; Nrf2: nuclear factor erythroid 2-related factor 2; NH_3_: ammonia; HG: high glucose; OGD/R: oxygen-glucose deprivation/reoxygenation; SMCs: smooth muscle cells; SOD: superoxide dismutase; tMCAO: transient middle cerebral artery occlusion.

## Abbreviations

AIS: Acute ischemic stroke
ankrd52: Ankyrin repeat domain 52
ANRIL: Antisense noncoding RNA in the INK4 locus
BPTF: Bromodomain PHD finger transcription factor
ceRNAs: Competitive endogenous RNAs
circRNAs: Circular RNAs
ciRNAs: Circular intronic RNAs
CMVECs: Cardiac microvascular endothelial cells
DLGAP4: DLG-associated protein 4
ecircRNA: Exonic circRNA
ecircRNAs: Exonic circular RNAs
EIciRNAs: Exon-intron circular RNAs
FBXW7: F-box and WD repeat domain containing 7
Foxo3: Forkhead box O3
GPx: Glutathione peroxidase
H_2_O_2_: Hydrogen peroxide
HECTD1: HECT domain E3 ubiquitin-protein ligase 1
HG: High glucose
HIPK3: Homeodomain interacting protein kinase 3
H/R: Hypoxia/reoxygenation
HUVEC: Human umbilical vein endothelial cell
HUVEC-Exos: Human umbilical vein endothelial cell exosomes
ICH: Intracerebral hemorrhage
I/R: Ischemia-reperfusion
IRI: Ischemia-reperfusion injury
ITCH: Itchy E3 ubiquitin-protein ligase
lncRNAs: Long noncoding RNAs
MBL: Muscleblind
miRNAs: MicroRNAs
Nrf2: Nuclear factor erythroid 2-related factor 2
^1^O_2_: Singlet oxygen
O_2_^−^: Superoxide anion
O_3_: Ozone
OGD/R: Oxygen-glucose deprivation/reoxygenation
OH^−^: Hydroxyl radicals
OXR1: Antioxidant 1
PABPN1: Poly(A) binding protein nuclear 1
PRKCI: Protein kinase C iota
RBPs: RNA binding proteins
ROS: Reactive oxygen species
SHPRH: SNF2 histone linker PHD RING helicase
SH-SY: Human neuroblastoma
SLC8A1: Solute carrier family 8 member A1
snRNPs: Small nuclear ribonucleoproteins
SOD: Superoxide dismutase
tMCAO: Transient middle cerebral artery occlusion
VSMCs: Vascular smooth muscle cells
ZNF609: Zinc finger protein 609.
